# Characterization of the biodistribution profile of a human Dialyzable Leukocyte Extract (hDLE) by *in vivo* fluorescence imaging: a strategy to infer the ADME profile of complex multipeptide drugs

**DOI:** 10.3389/fphar.2025.1701647

**Published:** 2026-01-12

**Authors:** Ana Fragozo, Ismael Trejo-Martínez, Kuauhtémok Domínguez-Bernal, Luis Valencia-Flores, Zaira Macias-Palacios, Lenin Pavón, Armando Pérez-Torres, Francisco A. Aguilar-Alonso, Said Vázquez-Leyva, Luis Vallejo-Castillo, Sonia Mayra Pérez-Tapia

**Affiliations:** 1 Unidad de Desarrollo e Investigación en Bioterapéuticos (UDIBI), Escuela Nacional de Ciencias Biológicas, Instituto Politécnico Nacional, Mexico City, Mexico; 2 Laboratorio Nacional para Servicios Especializados de Investigación, Desarrollo e Innovación (I+D+i) para Farmoquímicos y Biotecnológicos (LANSEIDI-FarBiotec-CONACyT), Escuela Nacional de Ciencias Biológicas, Instituto Politécnico Nacional, Mexico City, Mexico; 3 Departamento de Inmunología, Escuela Nacional de Ciencias Biológicas, Instituto Politécnico Nacional, Mexico City, Mexico; 4 Laboratorio de Psicoinmunología, Dirección de Investigaciones en Neurociencias, Instituto Nacional de Psiquiatría Ramón de la Fuente, Mexico City, Mexico; 5 Departamento de Biología Celular y Tisular, Facultad de Medicina, Universidad Nacional Autónoma de México, Mexico City, Mexico

**Keywords:** biodistribution, human dialyzable leucocyte extract, oral peptides, peptide gastric absorption, Transferon Oral^®^

## Abstract

The immunomodulator Transferon Oral^®^ (TO) is a human Dialyzable Leukocyte Extract (hDLE), *id est*, a complex mixture of peptides smaller than 10 kDa derived from human buffy coats. TO reduces TNF-α, IL-6, and IgE; and induces IFN-γ levels, which makes it useful in the treatment of allergies, autoimmunity, and infections. Its complex composition has made it difficult to characterize its pharmacokinetic profile, target organs, and mechanism of action. This study focused on describing the biodistribution profile of TO peptides indirectly using *in vivo* imaging. The TO peptides were coupled to the fluorophore Alexa 488 and administered intravenously (IV), subcutaneously (SC), intraperitoneally (IP), intramuscularly (IM), and oropharingelly (ORO) to female and male Crl: Nu-Foxn1nu Nu/Nu nude mice. It was verified that the TO peptides linked to the fluorophore maintained their biological activity (increment of survival in a murine model of HSV-1 infection) as a critical quality control. The fluorescence was acquired using an IVIS imaging system. Parenteral administration routes showed consistent biodistribution of TO peptides from the site of administration to the cervical and axillary areas, except for the IM route, where no biodistribution pattern was observed. The IV route of administration reached maximum fluorescence the fastest, at 15 min, and the peptides remained in the study subjects for around 180 min in all routes. Interestingly, the TO peptides were absorbed *via* ORO and exhibited a biodistribution pattern similar to that of the parenteral routes. Elimination *via* glomerular filtration was observed for all administration routes, and accumulation of TO peptides in the axillary and lymph nodes, as well as the heart, was confirmed by *ex vivo* analysis. The results of this work are relevant because they identify how the peptide components of an hDLE are absorbed enterally and parenterally and consistently accumulate in immunologically relevant organs where they exert their immunomodulatory function. Furthermore, it sets the first precedent for indirectly describing the absorption, distribution, metabolism, and excretion processes of a drug composed of a myriad of molecules using *in vivo* imaging.

## 1 Introduction

Therapeutic use of proteins and peptides has gained relevance in medicine, highlighting their advantages, such as higher selectivity and lower toxicity, compared to small drugs (La Manna et al., 2018; Wang et al., 2022). Furthermore, peptides, owing to their small size (usually less than 5,000 Da), are less immunogenic, and their cost of production is low compared to proteins (Wang et al., 2022; Dimitrov, 2019). Despite these advantages, therapeutic peptide development faces challenges, such as rapid degradation and quantification in biological samples, especially those with an endogenous sequence or those complex drugs composed of a myriad of components.

Dialyzable Leukocyte Extracts (DLE) are complex mixtures of low-molecular-weight peptides (usually smaller than 12 kDa), a high content of glycine residues, osmolarity values of 520 ± 90 mosm/L, and an OD_260_/OD_280_ ratio of 1.8–3.0 (Arnaudov and Kostova, 2015). The peptidic components of the DLE are obtained by filtration or dialysis of previously lysed buffy coats obtained from healthy humans (Medina-Rivero et al., 2014). DLE may be considered complex drugs according to the criteria of the Food and Drug Administration (FDA), considering that their Active Pharmaceutical Ingredient (API) is a mixture of different components with a distribution of molecular weight, thus limiting the complete characterization of their mechanism of action (MoA) or pharmacokinetic profile (FDA, CDER. MAPP 5240.10, 2022; Zagalo et al., 2022). Despite their complexity, DLEs have shown immunomodulatory activities (Arnaudov and Kostova, 2015) when used as auxiliary treatments for allergies (Flores Sandoval et al., 2005), infections (Estrada-Parra et al., 1998), autoimmune diseases (Lawrence, 1965) and cancer (Demečková et al., 2017; Hernández-Esquivel et al., 2018).

Transferon Oral^®^ (TO) is an hDLE whose API comprises peptides smaller than 10 kDa and monomeric Ubiquitin (Vallejo-Castillo et al., 2020). The peptide mixture of TO is highly hydrophilic, has a Polydispersity Index (PDI) by Mass Spectrometry of 1.28, and its most abundant amino acid residues are Gly (18.30%), Glu (14.49%), and Ala (11.53%) (Medina-Rivero et al., 2014). Further, the Size Exclusion Chromatography (SEC), Reversed Phase Ultra High-Performance Chromatography with UV and MS detection (RP-UHPLC-UV/MS), and water presaturation proton NMR spectra of TO are highly reproducible among batches (Herbert-Pucheta et al., 2021; Medina-Rivero et al., 2014, 2016).

Preclinical research has shown that TO modulates the inflammatory response in infections. In the herpes simplex virus-1 (HSV-1)-infected mice model, TO increases survival when orally administered by promoting the production of the antiviral cytokine IFN-γ and decreasing plasma levels of the proinflammatory molecules TNF-α and IL-6 (Salinas-Jazmin et al., 2015). On the other hand, Muñoz et al. (2021) studied the effect of adding Transferon Oral^®^ to the conventional treatment in puppies with systemic inflammatory response syndrome (SIRS) due to canine parvovirus (CPV) infection. In that study, the CPV-infected puppies co-treated with subcutaneous TO kept the Neutrophile Leucocyte Ratio (NLR) levels low, which in turn improved the survival rate and the clinical state compared to those receiving only the conventional treatment (Muñoz et al., 2021; Muñoz et al., 2022). In humans, oral administration of TO has been used to improve symptoms in patients with allergic rhinitis and atopic dermatitis (Homberg et al., 2015; Homberg et al., 2023) and in infections such as herpes zoster (Estrada-Parra et al., 1998; Flores Sandoval et al., 2005). In addition, in a retrospective study involving pediatric patients with sepsis, the co-administration of TO was associated with lower levels of C-reactive protein, an increase in lymphocyte count, and a decrease in neutrophil count, which influenced a higher survival rate (Castrejón Vázquez et al., 2019).

Although the above set of reports proves the efficacy of TO, its intrinsic complexity and the lack of pharmacokinetic studies limit the characterization of its MoA and the obtaining of key information to understand how orally administered peptides can induce a systemic immunomodulatory effect. However, as a first step to delve into its MoA, Vallejo-Castillo et al. (2020) identified the sequence of the peptidic components of TO using mass spectrometry. They found that the main components are peptides derived from 12 clusters of human proteins: ankyrin-1, protein 4.1, hemoglobin-α, and -β, Complement-3 protein, calpastatin, α-synuclein, fibrinogen α-chain, actin, thymosin, and Zyxin, along with full-length monomeric Ubiquitin (Ub^1-76^) and a Ub variant without the two terminal Gly (Ub^1-74^) (Vallejo-Castillo et al., 2020).

Another challenge in the preclinical research of TO is characterizing its absorption, biodistribution, metabolism, and excretion (ADME) processes. The ADME process of a complex drug, such as TO, cannot be determined by a classical pharmacokinetic profile due to its myriad of components; it is generally assumed that the metabolism of an oral peptide biodrug should be similar to the catabolism of dietary peptides and proteins. However, the characterization of the ADME profile could provide key information about the target tissues of TO and whether its biological effect occurs in the gastrointestinal tract or outside of it.

In this sense, this work aimed to infer the ADME process of TO by characterizing the biodistribution of its peptidic components through fluorescence imaging in mice as an indirect way to partially characterize its PK profile. To this end, the TO peptides were covalently linked to the Alexa Fluor 488 fluorophore, and their fluorescence kinetics were determined in Nu/Nu nude mice using oral and parenteral administration routes. The maximum fluorescence time was determined for each administration, and the tissues to which the peptides were biodistributed were identified based on the administration route with the best distribution profile.

## 2 Materials and methods

### 2.1 Human Dialyzable Leukocyte Extract

The human Dialyzable Leukocyte Extract (hDLE) used in this work was Transferon Oral^®^ (Batch 19K25), hereafter referred to only as hDLE. The hDLE was manufactured by Pharma-FT Laboratories (Mexico City; Mexico) using a previously reported process (Medina-Rivero et al., 2016). Briefly, 450 Buffy coats were collected from certified blood banks, and the cell content was lysed by freeze/thaw cycles. Peptides smaller than 12 kDa were obtained by filtration; subsequently, peptides smaller than 10 kDa were purified by tangential filtration. The peptide concentration of the hDLE was determined by UV absorption, adjusted to 0.4 mg/mL with injectable water, and stored in 10-mL high-density polypropylene vials at −20 °C until use. The hDLE batch met the following quality control analysis before use: sterility, identity (characteristic profile of eight peptide populations), pH (6.0–8.0), endotoxin content (≤4.0 UE/mL), relative density (1.0000–1.100), total protein content (0.4 mg/mL ± 15%), and biological potency in Jurkat Cells (80%–125%) (Carballo-Uicab et al., 2019; Medina-Rivero et al., 2016; Vallejo-Castillo et al., 2020).

### 2.2 Mice

Male Balb/C mice aged 4–6 weeks (Ferandelh; Mexico City, Mexico) and male and female Crl: Nu-Foxn1^nu^ Nu/Nu nude mice aged 4–6 weeks (UPEAL-Cinvestav; Mexico City, Mexico) were used in this work. Mice were housed in a P/NC IVC system (Allentown Inc., NJ, USA) at 22 °C and 55% relative humidity in pathogen-free conditions. Mice were subjected to a standard light-dark cycle and had *ad libitum* access to standard rodent chow (Lab Diet; MO, USA Cat. 5010). All procedures involving animals were performed in accordance with the Federal Regulations for the Use and Care of Animals (Secretaría de Agricultura, 1999) issued by the Mexican Ministry of Agriculture, as well as the International Guiding Principles for Biomedical Research Involving Animals (Council for International Organizations of Medical Sciences and the International Council for Laboratory Animal Science, 2012). The experimental procedures were approved by the Research Ethics Committee of the National School of Biological Sciences at the National Polytechnic Institute in Mexico City (code number ZOO-008-2021).

### 2.3 Labeling of the hDLE peptides with Alexa Fluor 488

hDLE peptides were coupled to Alexa Fluor 488 on -NH_2_ residues using a commercial ALEXA-488 protein labeling kit (Thermo Fisher Scientific Pierce™; IL, USA Cat. A20181), according to the manufacturer’s instructions. Briefly, the hDLE sample was concentrated by lyophilization using a FreeZone Dry System (Telstar, PA, USA, Cat. LyoBeta-35), and 2 mg of lyophilized material was dissolved in 500 µL of 1 M sodium bicarbonate buffer, pH 9.0 (Solution A), to reach a final concentration of 4 mg/mL. On the other hand, the Alexa Fluor 488 dye was dissolved in 50 µL of sodium bicarbonate buffer (Solution B). Then, 15 µL of solution B was added to 500 µL of Solution A, and the mixture was incubated at room temperature for 4 h. The solution was added with 500 µL of 1X PBS and stored at 4 °C until use; the reaction mixture and the final conjugated solution were always protected from light.

### 2.4 Quality control of the hDLE-Alexa peptides

#### 2.4.1 Labeling confirmation of hDLE-Alexa peptides

The conjugation of the hDLE peptides to Alexa Fluor 488 (hDLE-Alexa peptides) was verified using Mass Spectrometry coupled to Reversed-Phase High-Performance Liquid Chromatography (RP-UPLC-MS). A 10-µL aliquot of labeled peptides was separated in an Acquity™ UPLC Class I system (Waters™; MA, USA) using a BioSuite C18 PA-A column (100 Å, 3 µm, 2.1 mm × 150 mm) (Waters^®^; MA, USA Cat. 186002427) at 60 °C, and a mobile phase composed of solution A [0.1% formic acid in water (Honeywell, NJ, USA Cat. 39253)] and solution B [0.1% formic acid in acetonitrile (Thermo Fisher Scientific Pierce™; IL, USA Cat. 51101)]. The mobile phase was infused at 0.2 mL/min as follows: 100% solution B from 0 to 10 min, then a linear gradient was applied from 100% to 50% solution B over 95 min. The chromatographic system was returned to initial conditions and stabilized for 10 min before the next injection. The components of the sample were analyzed using a Vion™ ESI IMS Q-ToF Mass Spectrometer at positive polarity and MS^E^ mode, 5.00 eV/10.00 eV of low/high collision energy, and 35.00 eV of high collision energy ramp end. The ionization source was set at 150 °C temperature, 450 °C desolvation temperature, 0 L/h cone gas, 1.000 L/h desolvation gas, and 2.75 kV capillary voltage. Data were acquired from 50 to 2000 m/z and adjusted using a 50-pg/mL Leucine Enkephalin standard solution (Waters^®^, MA, USA, Cat. 186006013) infused at 5 μL/min. The MS data were processed using the UNIFI™ software (Waters™; MA, USA).

#### 2.4.2 Verification of biological activity of hDLE-Alexa peptides

A murine model of HSV-1 infection was used to confirm that the hDLE peptides maintain their biological activity after binding to Alexa Fluor 488. The assay was performed as described by Salinas-Jazmin et al. (2015). Briefly, 4-week Balb/C mice weighing 14–18 g were anesthetized by intraperitoneal administration of 64 μg/kg body weight of sodium pentobarbital (Laboratorios Pisa, Jalisco, Mexico, Q-7833-215) and 1 cm^2^ of dorsal skin was depilated. Immediately after, mice were infected with 10-µL of 1.1 × 10^8^ plaque-forming units (PFU)/mL of HSV-1 (KOS strain ATCC, VR-934) by scarification. HSV-1-infected animals were distributed in five groups (*n* = 10 per group) and orally administered with 0.750 µg of native hDLE (Group 1), hDLE-Alexa (Group 2), and lyophilized and reconstituted hDLE (lyophilization control) (Group 3); or Alexa Fluor 488 alone, equivalent to the amount used to label the hDLE peptides (fluorophore control) (Group 4), and water for injection (infection control) (Group 5). An observational control (not infected/not treated group) was also included. Treatments were administered on days 2, 4, 6, 8, and 10 post-infection. The mice were monitored until day 20 post-infection to identify signs associated with HSV-1 infection, such as paralysis in the lower extremities, cachexia, kyphosis, decreased mobility, and weight loss; animals showing any of these signs were euthanized and counted as deaths for survival analysis.

### 2.5 Kinetic biodistribution of hDLE-Alexa

Male and female Nu/Nu Nude mice were fasted for 4 h. Then, animals were anesthetized with inhaled isoflurane (Laboratorios Pisa, Jalisco, Mexico, Cat. Q-783322) using an anesthesia chamber (5% for induction and 0.5%–1% for maintenance) in O_2_ at a flow rate of 1 L/min. Next, 100 µL of hDLE-Alexa (100 µg total protein per dose) was administered oropharyngeally (ORO), subcutaneously (SC), intramuscularly (IM), intraperitoneally (IP), or intravenously (IV) (*n* = 4 per group); an Alexa Fluor 488 control was included per *via*. Fluorescence was monitored using the IVIS LUMINA XR system (Caliper Life Science, MA, USA). The excitation wavelength was set at 465 nm, with the 510-emission filter, automatic exposure time, and a height of 12 cm. Dorsal and ventral views were acquired at 15, 30, 60-, 90-, 120,150-, and 180-min post-administration using the Living Image^®^ 4.3.1 software (Caliper Life Science, MA, USA). The autofluorescence (AF) signal of each mouse was recorded before the administration of Alexa Fluor 488 or hDLE-Alexa.

### 2.6 *Ex vivo* biodistribution analysis of hDLE-Alexa after ORO administration

The biodistribution assay was replicated to determine the accumulation of hDLE-Alexa peptides in mouse tissues following ORO administration. To this end, 100 µg of hDLE-Alexa was administered to male and female nude mice (*n* = 4 per sex), which were then euthanized at the time of maximum fluorescence, as determined by ORO kinetic assays (60 min). The following organs were then extracted: spleen, axillary and cervical lymph nodes, intestine, stomach, liver, kidneys, heart, and lungs. The cervical and axillary lymph nodes were identified and extracted based on their anatomical characteristics, as shown in Supplementary Figure S1. Tissue fluorescence was acquired using the same parameters as for the whole animal, and to expedite the analysis, the organs were grouped and analyzed as follows: cervical and axillary lymph nodes (group 1); liver, intestine, and stomach (group 2); and kidneys, heart, lungs, and spleen (group 3). We used as control organs of mice treated with Alexa alone and euthanized at the same post-administration time (60 min). Control animals were administered 100 μL of an Alexa solution prepared exactly as described in the labeling peptides section, but using 1X PBS instead of the hDLE solution.

### 2.7 Image processing

Whole mouse fluorescence was recorded after administration of Alexa Fluor 488 or the hDLE-Alexa peptides and processed using the Living software Image^®^ version 4.3.1. The images of all the mice were divided into four regions of interest (ROI) for the analysis, corresponding to the head-neck, chest, abdomen, and genitourinary areas, as shown in Supplementary Figure S2. Autofluorescence ROIs (images acquired before administering Alexa Fluor 488 or hDLE-Alexa) were subtracted from ROIs obtained during the biodistribution kinetics. The fluorescence was reported in the average radiant efficiency of ROIs [p/s]/[μW/cm^2^].

### 2.8 Statistical analysis

All statistical analyses were conducted using GraphPad Prism software (version 10.0.0 for Windows; GraphPad Software, San Diego, CA, USA). Survival rates in the murine HSV-1 infection model were analyzed using Kaplan-Meier survival curves, with statistical significance determined by the log-rank (Mantel-Cox) test. The Shapiro-Wilk test was applied to assess normality for organ epifluorescence and mean fluorescence intensity (MFI) across different administration routes. Inter-group comparisons were performed using Welch’s ANOVA for unequal variances, followed by the Dunnett T3 *post hoc* test for multiple comparisons. Data are presented as mean ± standard error of the mean (SEM). A *p*-value <0.05 was considered statistically significant.

## 3 Results

### 3.1 Labeling hDLE peptides with Alexa Fluor 488 does not affect their biological activity

**FIGURE 1:**
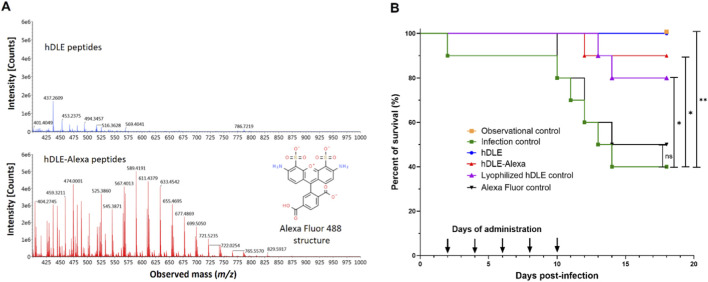
The hDLE peptides maintain their biological activity after conjugation with Alexa Fluor 488. **(A)** The m/z mass spectrometry profile of hDLE (blue spectra) and hDLE-Alexa (red spectra) showed that the ionization increases after conjugation because hDLE peptides acquire the ionization groups of Alexa Fluor 488 (*in set*), such as two -NH_2_. **(B)** The activity of hDLE-Alexa peptides was tested in a murine HSV-1 infection model. Observational (not infected/not treated) and infection (infected/not treated) controls showed 100% and 40% survival, respectively. hDLE, hDLE-Alexa, and the lyophilization hDLE control showed a Δ% survival rate of ≥40% compared to the infection control. All treatments were orally administered (0.75 μg) five times per mouse after HSV-1 infection. An additional infected group was orally administered with Alexa Fluor 488 (Alexa Fluor control), and no statistical differences were observed compared to the infection control. The Kaplan-Meier and *post hoc* Log-rank (Mantel-Cox) tests were performed to determine statistical significance; *p ≤ 0.05, **p < 0.01; *n* = 10.

The first step of this work was conjugating the hDLE peptides to Alexa Fluor 488. The hDLE peptides were concentrated by lyophilization and covalently linked to Alexa Fluor 488 in sodium bicarbonate buffer (pH 9.0); the conjugation was verified using RP-UHPLC-MS. All the data acquired during the chromatographic analysis of each sample were combined to obtain the *m/z* profile from 400 to 1,000 m/z using the software UNIFI™. As shown in Figure 1A, the ionization profile of the hDLE-Alexa presents a higher signal intensity and a more significant number of m/z signals than the non-conjugated hDLE peptides, owing to the addition of ionizable groups (-NH_2_) from the Alexa Fluor 488 molecule.

The addition of a fluorophore to the structure of hDLE peptides could alter their physicochemical and biological properties. Therefore, it was verified that hDLE-Alexa peptides maintained their biological activity (increased survival) in the mouse model of HSV-1 infection. In this assay, it is expected that mice infected with HSV-1 and orally treated with hDLE present an increase in survival by 40% (≥Δ40%) compared to the infection control (Salinas-Jazmin et al., 2015). As shown in Figure 1B, infection control and observational control showed 40% and 100% survival, respectively, whereas the hDLE and hDLE-Alexa peptides induced 90% (Δ50%; *p* ≤ 0.05) and 100% (Δ60%; *p* ≤ 0.01) survival, respectively, both exceeding the acceptance criterion of the test. On the contrary, the Alexa Fluor control induced 50% survival (Δ10%; statistically not significant) compared to the infection control. In addition, since the hDLE was concentrated by lyophilization before conjugation, we included an HSV-1-infected mice group treated with a hDLE that underwent a lyophilization cycle (lyophilization control), which also met the acceptance criteria test (Δ 40%; *p* ≤ 0.05). Differences between the hDLE-Alexa and hDLE-treated groups, and between the hDLE-lyophilized and hDLE-treated groups, were not statistically significant (*p* > 0.05). In general, dyes used in *in vivo* imaging are low-toxicity agents, and for Alexa Fluor 488, no toxicity data have been reported (Alford et al., 2009). Although a control group of healthy mice administered only Alexa Fluor 488 was not evaluated, the results of the survival assay corroborate the fluorophore’s low toxicity and do not affect the biological activity of the hDLE peptides.

### 3.2 Biodistribution of hDLE-Alexa peptides after IV administration

**FIGURE 2:**
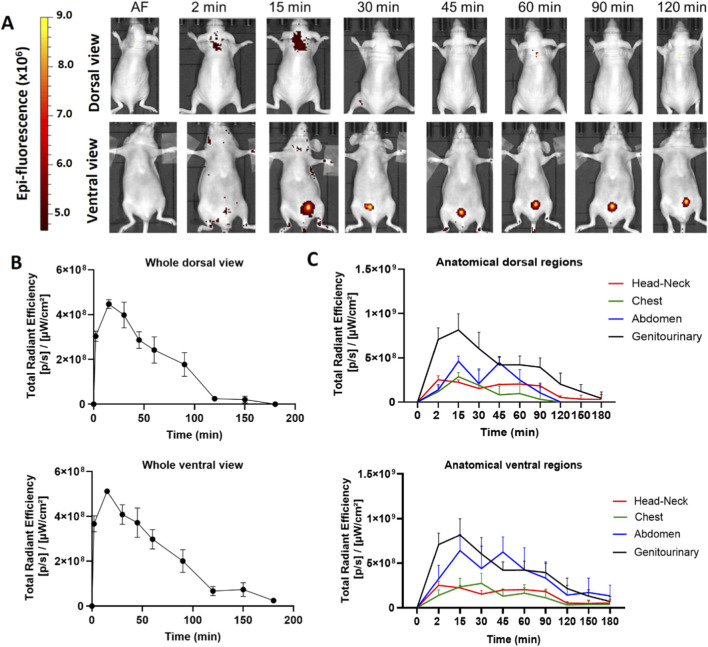
The hDLE-peptides are distributed to the head-neck and genitourinary area of female Nu/Nu nude mice after IV administration. The hDLE-Alexa peptides were administered in four female Nu/Nu mice in the tail vein, and epifluorescence was detected using an IVIS LUMINA XR system. **(A)** Representative dorsal and ventral images of the same animal showing the biodistribution of the hDLE-Alexa peptides during 120 min; it is observed that hDLE-Alexa peptides are biodistributed to the neck and the genitourinary areas. **(B)** Total Radiant Efficiency plots of the dorsal and ventral accumulation fluorescence kinetics of the four administered mice; the maximum signal is observed at 15 min post-administration. **(C)** Total Radiant Efficiency of the hDLE-Alexa peptides per specific anatomical area of the four administered mice (genitourinary, abdominal, chest, and head-neck) after IV administration; the genitourinary area exhibits the maximum epifluorescence values, both in dorsal and ventral views. The plots represent the average radiant efficiency of all selected ROIs (whole or a specific region) vs. time from dorsal and ventral images of the four analyzed animals.

We determined the biodistribution profile of the hDLE-Alexa peptides after IV administration in anesthetized Nu/Nu nude mice. We used this strain of mice because the hair fluoresces naturally, and hairlessness allows us to reduce the background and easily visualize the signal of the labeled peptides. After IV administration in female Nu/Nu nude mice, the epifluorescent signal of hDLE-Alexa peptides appears at 2 min, reaches the maximum intensity at 15 min in the neck and the genitourinary region (bladder), and decreases to basal values at 180 min, as shown in heat images and the Total Radiant Efficiency plot in Figures 2A,B, respectively. When analyzing the biodistribution by anatomical region at 15 min post-administration, it was observed that fluorescence accumulation follows this order: genitourinary < abdomen < chest and head-neck region, in both dorsal and ventral views, as shown in Figure 2C. The biodistribution profile of hDLE-peptides was similar in male Nu/Nu nude mice, although the fluorescence signals remained up to 180 min, as shown in Supplementary Figure S3.

### 3.3 Biodistribution of hDLE-Alexa peptides after SC administration

**FIGURE 3:**
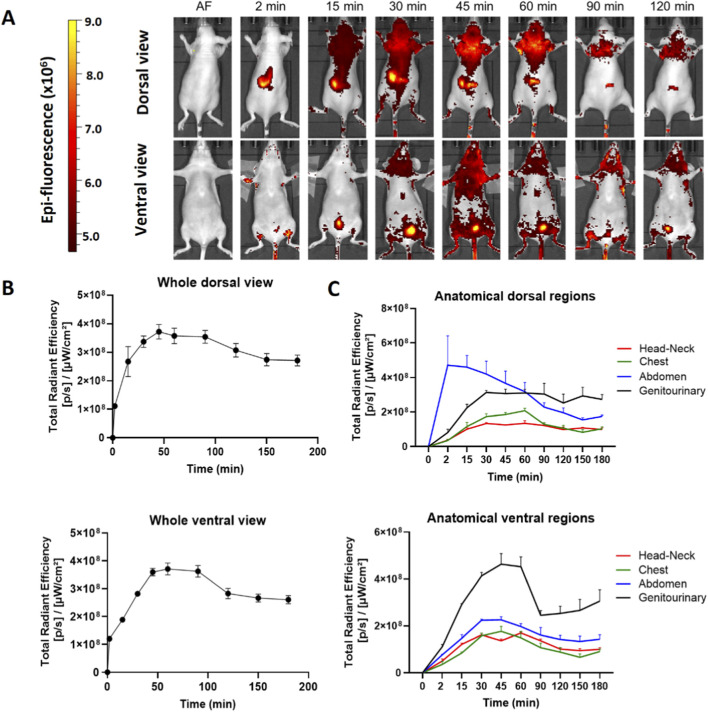
hDLE-Alexa peptides are widely biodistributed and accumulated in the head-neck and genitourinary area of Nu/Nu nude female mice after SC administration. The hDLE-Alexa peptides were administered in four female Nu/Nu mice in the back, and epifluorescence was detected using an IVIS LUMINA XR system. **(A)** Representative dorsal and ventral images showing the biodistribution of the hDLE-Alexa peptides during 120 min; it is observed that the peptides are widely biodistributed in all anatomical regions of the mice. The 2-min dorsal view evinced the site of administration of the hDLE-Alexa peptides. **(B)** Total Radiant Efficiency plots of the dorsal and ventral accumulation fluorescence kinetics of the four administered mice; the maximum signal is observed around 45–60 min post-administration. **(C)** Total Radiant Efficiency of the hDLE-Alexa peptides per specific anatomical area of the four administered mice (genitourinary, abdominal, chest, and head-neck) after SC administration; in the dorsal view, the region of greatest fluorescence is the abdomen, while in the ventral view it is the genitourinary area. The plots represent the average radiant efficiency of all selected ROIs (whole or a specific region) over time, as determined from dorsal and ventral images of the four analyzed animals.

We obtained the distribution profile of the hDLE-Alexa peptides when administered subcutaneously (SC) in the back of female Nu/Nu nude mice. After SC administration, the hDLE-Alexa peptides moved from the site of administration to the head-neck, the chest, and the abdomen area in the dorsal view within 15 min, and the head-neck, the chest, and the genitourinary area in 15–30 min in the ventral view, as shown in Figure 3A. The total radiant efficiency plot shows that fluorescence reached maximum values at 45 min (dorsal view) and 60 min (ventral view) after administration, as observed in Figure 3B. The biodistribution surface of hDLE-Alexa peptides was greater after SC administration compared to IV administration. However, as time progressed, it became evident that the accumulation sites of hDLE peptides are similar for both routes of administration: the neck area in both the ventral and dorsal views, and in the genitourinary (bladder) in the ventral view. In addition, in SC administration, an accumulation of hDLE peptides is observed in the inguinal area. When analyzing the biodistribution by anatomical region, it was observed that fluorescence accumulation occurs in the following order: abdomen > genitourinary > chest > head & neck region in the dorsal view, whereas genitourinary > abdomen > chest > head & neck region in the dorsal view, as shown in Figure 3C. An accumulation phenomenon is observed from 0 to 60 min and from 60 to 180 min in the genitourinary area (ventral view), which correlates with the accumulation of urine in the bladder. After 180 min of monitoring, the fluorescence signal did not reach the baseline in the females administered with hDLE-Alexa. The assay was replicated in male Nu/Nu mice, and the same biodistribution patterns were observed, as shown in Supplementary Figure S4. Notwithstanding, there were some differences: i) the fluorescence signal returns to the baseline after 180 min post-administration, ii) a higher inter-individual variation is observed, and iii) the last area to maintain fluorescence is the administration site, whereas in females it is the genitourinary zone.

### 3.4 Biodistribution of hDLE-Alexa peptides after IP administration

**FIGURE 4:**
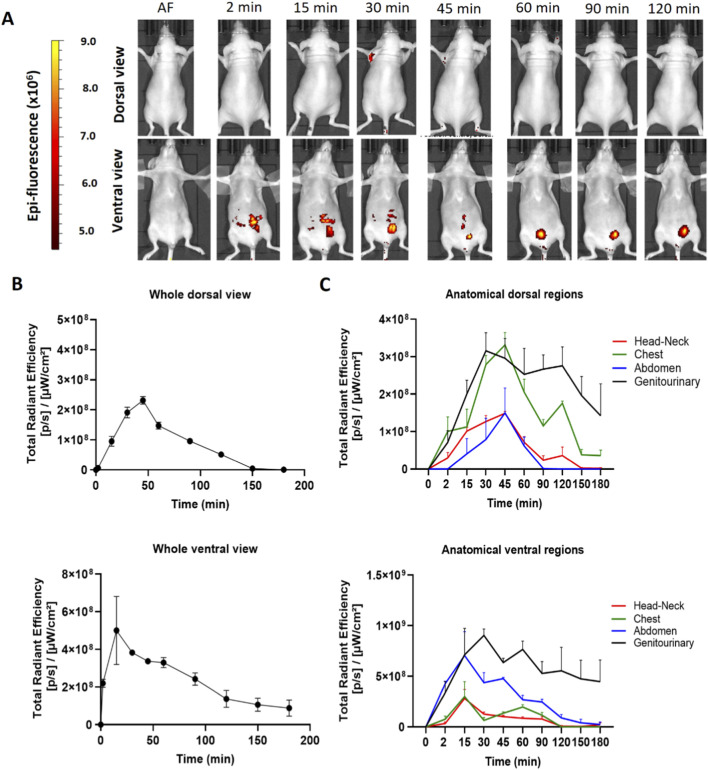
hDLE-Alexa peptides are poorly biodistributed in Nu/Nu nude female mice after IP administration. The hDLE-Alexa peptides were intraperitoneally administered in four female Nu/Nu mice, and epifluorescence was detected using an IVIS LUMINA XR system. **(A)** Representative dorsal and ventral images showing the biodistribution of the hDLE-Alexa peptides during 120 min; it is observed that the peptides are poorly biodistributed from the administration site to the axillary area. The 2-min ventral view evinced the site of administration of the hDLE-Alexa peptides. **(B)** Total Radiant Efficiency plots of the dorsal and ventral accumulation fluorescence kinetics of the four administered mice; the maximum signal is observed around 45 and 15 min in the dorsal and ventral view, respectively. **(C)** Total Radiant Efficiency of the hDLE-Alexa peptides per specific anatomical area of the four administered mice (genitourinary, abdominal, chest, head-neck) after IP administration. In the dorsal view, the region of greatest fluorescence is the genitourinary and chest areas, while in the ventral view, it is the genitourinary area. The plots represent the average radiant efficiency of all selected ROIs (whole or a specific region) over time, as determined from dorsal and ventral images of the four analyzed animals.

The hDLE-Alexa peptides were administered *via* IP in female Nu/Nu nude mice. As shown in Figure 4A, the hDLE-Alexa peptides remained in the administration area (ventral) from 2 to 45 min, where a cyclic accumulation was also observed in the bladder throughout the monitoring. Regarding the dorsal view, a slight biodistribution is seen in the axillary area around 30 min post-administration. Figure 4B shows that the maximum peak of fluorescence is observed at 45 min in the dorsal view and at 15 min in the ventral view. In this sense, Figure 4C illustrates that the ventral view exhibits the maximum fluorescence in the genitourinary area, which is composed of the fluorescence signal at the administration site that disappears during monitoring, as well as the cyclical fluorescence in the bladder due to the accumulation and emptying of urine.

The biodistribution of hDLE-Alexa peptides was also evaluated in male Nu/Nu nude mice intraperitoneally. As seen in Supplementary Figure S5, hDLE-Alexa peptides were biodistributed from the administration site to the head and neck area, in both dorsal and ventral views. Additionally, it is observed that the fluorescence signal disappears from the administration site before 120 min in the ventral view, and fluorescence is also accumulated in the bladder region. In male mice, the maximum fluorescence values are observed at 45 min and 60 min in the genitourinary area in both dorsal and ventral views, respectively.

### 3.5 Biodistribution of hDLE-Alexa peptides after IM administration

**FIGURE 5:**
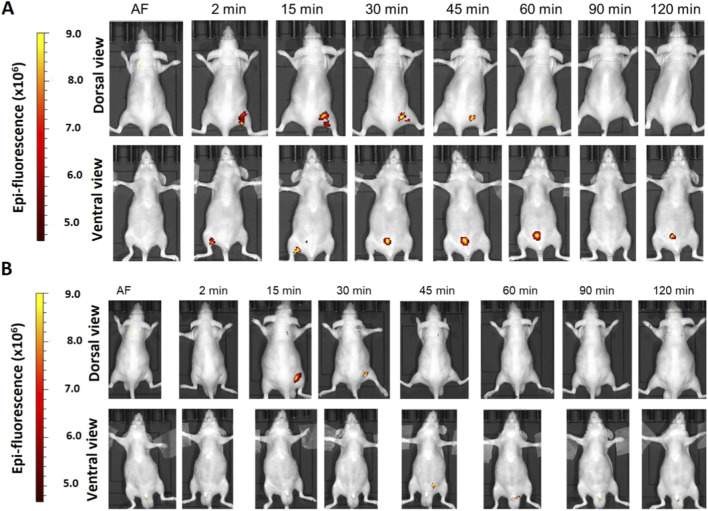
A biodistribution profile of hDLE-Alexa peptides is not observed after IM administration in female and male Nu/Nu nude mice. The hDLE-Alexa peptides were administered in four female and four male Nu/Nu mice in the dorsal part of the right rear paw, and epifluorescence was detected using an IVIS LUMINA XR system. **(A)** The fluorescence signal is observed in the administration site from 2 to 45 min in female mice, whereas from 15 to 30 min in male mice. The site of administration is observed in the 2-min dorsal view. **(B)** Biodistribution monitoring of hDLE-Alexa peptides in male nude mice. The site of administration is observed in the 15-min dorsal view. Accumulation of hDLE-Alexa peptides is observed in the bladder of both sexes, although with more intensity in female mice.

Evaluation of parenteral administration of hDLE-Alexa peptides was completed with IM administration in both female and male Nu/Nu nude mice. The hDLE peptides were inoculated in the dorsal part of the right rear paw. As shown in Figure 5, the fluorescence signal of the inoculation site remained from 2 to 45 min in females and from 15 to 30 min in males; however, a biodistribution pattern was not observed in either sex. On the other hand, an accumulation phenomenon was observed in the bladders of both female and male animals, with a more pronounced occurrence in females. Since no biodistribution profile was observed using the IM route, Total Radiant Efficiency analysis was not calculated for the whole animal and individual areas.

### 3.6 Biodistribution of hDLE-Alexa peptides after ORO administration

**FIGURE 6:**
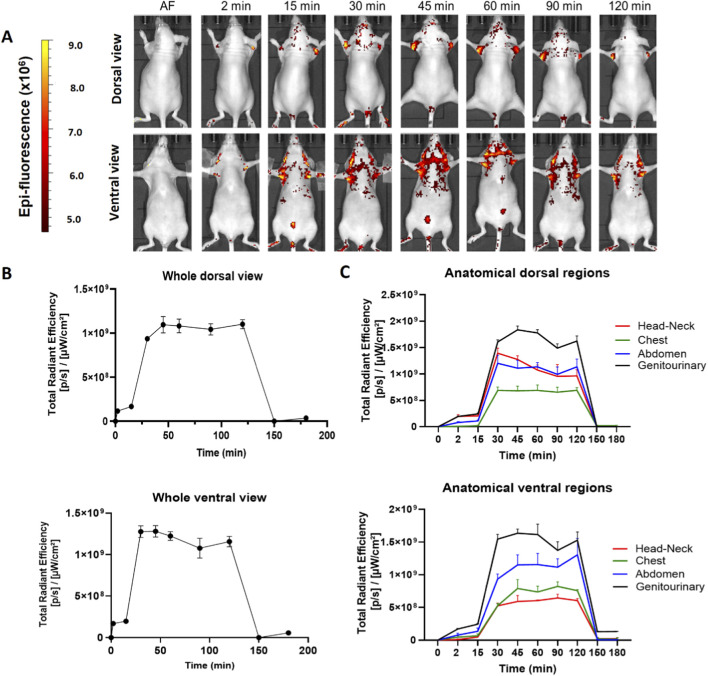
The hDLE-Alexa peptides are absorbed from the gastrointestinal tract and biodistributed to the head-neck and axillary area after oral administration in female Nu/Nu nude mice. Four female mice were administered hDLE-Alexa peptides *via* the ORO, and epifluorescence was detected using an IVIS LUMINA XR system. **(A)** Representative dorsal (top) and ventral (bottom) images showing the biodistribution of the hDLE-Alexa peptides over a 120 min period. **(B)** Global body accumulation kinetics of the hDLE-Alexa peptides after ORO administration in female mice. Plots show the average total radiant efficiency of Regions of Interest (ROI) over time (minutes) from complete dorsal (top plot) and ventral (bottom plot) images. **(C)** Biodistribution kinetics of the hDLE-Alexa conjugate in each specific anatomical area after ORO administration in female mice. Plots are the average of the total radiant efficiency of specific ROIs (genitourinary, abdominal, chest, and head-neck) vs. time (min) from dorsal (top plot) and ventral (bottom plot) images. Total Radial Efficiency ([p/s]/[μW/cm^2^]) was quantified in selected ROI from dorsal and ventral position images using the Live Image^®^ 4.3.1 software.

The hDLE used in this study modulates the levels of proinflammatory cytokines, interferon gamma, and the NRL ratio in preclinical models when orally and intraperitoneally administered (Salinas-Jazmin et al., 2015; Muñoz et al., 2022), and improves the quality of life in adult patients with allergic rhinitis when orally administered (Homberg et al., 2023). In this sense, we evaluated the oral administration of the hDLE-Alexa peptides *via* the oropharyngeal route (ORO) by introducing a metal cannula into the snout of the animals and depositing the drug in the esophagus. As shown in Figure 6A, the hDLE-Alexa peptides are absorbed from the gastrointestinal tract to the head-neck and axillary areas from 2 to 120 min after ORO administration in both ventral and dorsal views. In addition, a fluorescence signal is detected in the bladder area, as observed in parenteral administrations. The analysis of Total Radiant Efficiency reveals that the ORO administration of the hDLE-Alexa peptides exhibits a plateau-shaped profile, maintaining constant values from 15 to 150 min, both in the dorsal and ventral views, as shown in Figure 6B. We analyzed epifluorescence intensity by anatomical regions and found that the signal was higher in the genitourinary area in both dorsal and ventral views, as shown in Figure 6C.

Our results show that after ORO administration of hDLE-Alexa peptides, the intensity in females is greater than in males (1.09 × 10^9^ vs. 5.59 × 10^7^, respectively), as shown in Figure 6B and Supplementary Figure S6, respectively. In addition, in females, the fluorescence of hDLE-Alexa peptides returned to baseline values after 120 min, whereas in male mice, the signal persisted after 180 min. Table 1 summarizes the time and peak fluorescence intensity observed in the biodistribution profiles of hDLE-Alexa peptides in male and female nude mice.

**TABLE 1:** Summary of the fluorescence biodistribution profile of hDLE-Alexa peptides in Nu/Nu nude mice. The table summarizes the maximum fluorescence values detected and the time at which they were detected (Peak time), as well as the time at which the fluorescence signal reaches the baseline. The shown values correspond to the ventral view. The (*) indicates that the ventral and dorsal values are different; if this is the case, both values are also included (ventral/dorsal). The highest values among both sexes are marked in bold.

Via of administration	Biodistribution parameter	Female	Male
IV	Max. fluorescence ([p/s]/[μW/cm^2^])	5.12 × 10^8^	**1.26 × 10** ^**9**^
	Peak time (min)	15	15
	Return to baseline (min)	180	(*) > 180/180
SC	Max. fluorescence ([p/s]/[μW/cm^2^])	3.71 × 10^8^	2.71 × 10^8^
	Peak time (min)	60	(*) 60/15
	Return to baseline (min)	**>180**	180
IP	Max. fluorescence ([p/s]/[μW/cm^2^])	5.00 × 10^8^	**1.60 × 10** ^**9**^
	Peak time (min)	(*) 15/45	**60**
	Return to baseline (min)	150/180*	(*) 180/>180
IM	Max. fluorescence ([p/s]/[μW/cm^2^])	ND	ND
	Peak time (min)	ND	ND
	Return to baseline (min)	ND	ND
ORO	Max. fluorescence ([p/s]/[μW/cm^2^])	(*) 1.27 × 10 ^**9**^ **/1.10 × 10** ^**9**^	(*) 4.90 × 10^7^/5.59 × 10^7^
	Peak time (min)	(*) 30/45	(*) 30/120
	Return to baseline (min)	180	**> 180**

### 3.7 *Ex vivo* accumulation of hDLE-Alexa peptides after ORO administration

An *ex vivo* analysis was performed to identify tissues where potential accumulation of hDLE-Alexa peptides was observed. Considering the similar biodistribution profiles obtained, ORO administration was selected, as it is the preferred method of administration of hDLE in preclinical efficacy models. Additionally, the ORO route of administration showed the highest fluorescence intensity. Female nude mice were administered hDLE-Alexa peptides *via* ORO and euthanized at 60 min, considering that the maximum fluorescence observed in biodistribution kinetics occurred from 45 to 120 min. Then, the kidneys, axillary and cervical lymph nodes, lungs, heart, spleen, intestines, stomach, and liver were collected according to their macroscopic characteristics, as shown in Supplementary Figure S7. During the *ex vivo* analysis, we observed that autofluorescence intensity varied across organs from different animals. Thus, we considered the Alexa control (at a concentration equivalent to the hDLE-Alexa) to be the most appropriate control because allowed us to: a) obtain fluorescent signals well above baseline values (elimination of the autofluorescence effect) and b) identify TMDD phenomena that would guide us to discriminate between the organs that might play a more critical role in the immunologic mechanism of action of the DLE and the organs in which its biodistribution occurred in a non-specific way, such as the intestine, where Alexa Fluor 488 is known to be absorbed by intestinal cells (Michael Danielsen and Hansen, 2016), or organs with a high perfusion rate, such as the kidney, spleen, and liver.

**FIGURE 7:**
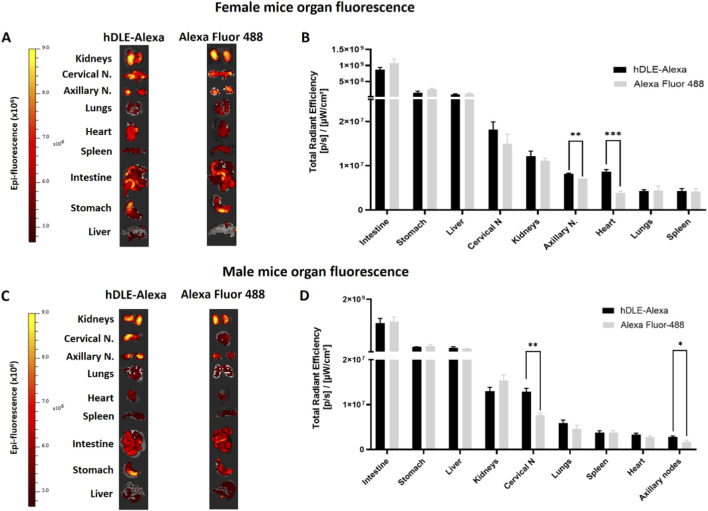
hDLE peptides are accumulated in axillary nodes, heart, and cervical nodes after ORO administration. For the accumulation analysis, nude male and female mice were administered either hDLE-Alexa or Alexa Fluor 488 (*n* = 4 per group) *via* ORO. Mice were euthanized 60 min post-administration and fluorescence values were obtained in the same way as in the kinetic analyses of hDLE in whole mice. **(A)** Images of the fluorescence of the organs of female nude mice extracted after 60 min of ORO administration of hDLE-Alexa peptides or Alexa Fluor 488 (control) and **(B)** histogram from respective Total Radial Efficiency. Panels **(C)** and **(D)** show the results obtained in a similar analysis performed on male nude mice. Note that all organ pictures were adjusted to a similar size for viewing and therefore do not maintain anatomical proportion, and that the fluorescence data in histograms is presented from highest to lowest fluorescence for each organ. When comparing the fluorescence of tissues from individuals treated with the peptides hDLE-Alexa peptides and Alexa Fluor 488, statistical differences were observed in the axillary nodes and the heart in the case of females, and in the cervical and axillary nodes in the case of male nude mice. A Shapiro-Wilk normality test and Welch’s ANOVA test were applied to determine statistical differences among the fluorescence of mouse organs; *p ≤ 0.05, **p < 0.01.

After administration, hDLE-Alexa peptides and Alexa Fluor 488 were highly detected in the intestines, the stomach, and the liver, as shown in Figures 7A,B. No statistically significant differences were found when the fluorescence intensity of the hDLE-Alexa and Alexa Fluor 488 peptides was analyzed per tissue, except in the axillary lymph nodes and the heart, where a higher signal was observed with the administration of hDLE-Alexa peptides. Additionally, the cervical lymph nodes exhibit a fluorescence intensity that tends to be higher in mice administered the hDLE-Alexa peptides. However, the variability of the signal obtained in this tissue does not allow a statistical conclusion. The test was replicated in male nude mice. As observed in Figures 7C,D, the fluorescence of the administered treatments was also detected more intensely in the intestine, stomach, and liver of male nude mice, as observed in female mice. On the other hand, a statistically significant difference was observed in the cervical and axillary lymph nodes of mice administered the hDLE-Alexa peptides compared to those that received Alexa Fluor 488 only. On the other hand, a trend was observed in the heart and lungs to exhibit greater fluorescence when administering hDLE-Alexa peptides, compared to controls administered only with Alexa Fluor 488.

These results reinforce the observation that hDLE-Alexa peptides are absorbed from the gastrointestinal tract and accumulate in the axillary lymph nodes in female nude mice. Additionally, accumulation is observed in the heart and cervical lymph nodes in female and male nude mice, respectively.

## 4 Discussion

Characterizing the pharmacokinetic (PK) profile during preclinical and clinical development of a new biodrug is essential for understanding its absorption, distribution, metabolism, and excretion (ADME) profile; correlating efficacy with plasma concentrations; and, in turn, establishing the dosage regimen (Conner et al., 2020). In addition, when investigating innovator biotherapeutic peptidic agents, the ADME profile is fundamental for identifying the target tissues and confirming their MOA, and identifying possible secondary effects (Silva Lima and Videira, 2018). It is worth mentioning that one of the critical requirements for determining the PK profile of any drug is to have a validated bioanalytical method to determine its concentrations in the biological matrix of interest, generally plasma.

The PK profile of peptides, as well as their ADME profile, is governed by two types of factors: their physicochemical properties and target-mediated drug disposition (TMDD) (Datta-Mannan, 2019). Among the intrinsic physicochemical characteristics of peptides that affect their PK profile are their size, structure, charge, hydrophobicity, post-translational modifications such as glycosylation, thermal stability, and catabolic stability (enzymatic degradation) (Datta-Mannan, 2019). On the other hand, the TMDD concept refers to a non-linear pharmacokinetic behavior, that is, the relationship between the administered doses and the concentrations identified in plasma is not proportional due to the high affinity of the biodrug for its therapeutic target (An, 2020). The challenge in determining the PK and ADME profile is greater in the case of complex multipeptide drugs, such as glatiramer acetate and hDLEs, which are composed of multiple peptides with distinct physicochemical and biological properties (Crommelin et al., 2015; Kasindi et al., 2022). Furthermore, hDLEs contain highly soluble peptides with natural sequences that cannot be distinguished from endogenous sequences (Vallejo-Castillo et al., 2020).

Multipeptide complex drugs require specific approaches to partially characterize their PK profile through biodistribution studies. Nowadays, there are several non-invasive imaging tools available to evaluate the biodistribution of therapeutic agents, such as Positron Emission Tomography (PET), X-ray Computed Tomography (CT), and Fluorescence Imaging (FI) and Targeted Fluorescent Live Imaging (TFLI), which have been utilized in preclinical applications due to their capability to capture high-quality, real-time images non-invasively (Yukawa and Baba, 2017). *In vivo* fluorescent live imaging, such as Fluorescence Molecular Tomography (FMT) and 2D-fluorescent imaging tomography (IVIS), is a common technique that offers advantages over radiation-based techniques because it is non-invasive, provides high resolution, and poses no radioactive risk (Giddabasappa et al., 2016; Yilmaz Usta et al., 2023). In addition, this versatile technique allows for real-time monitoring of the same subject over time, facilitating more efficient and cost-effective biodistribution studies (Van Oosten et al., 2013). For example, Gupta an cols. have evaluated different amine-reactive NIR fluorophores, such as Alexa Fluor 647^®^, Alexa Fluor 680^®^, and Alexa Fluor 750^®^, in preclinical studies of the biodistribution and tumor binding of an interleukin-13 receptor subunit alpha-2 antibody (IL13Rα2-Ab) in mice and cell lines (Gupta et al., 2017); Duygu and Cols. evaluated the oral biodistribution of bosentan (BOS), an oral non-peptidic endothelin receptor antagonist, formulated in a complex drug-delivery system using VivoTag^®^ 680XL for BOS and XenoLight™ DiR for the delivery system (Yilmaz Usta et al., 2023); and Xin and cols. Reported the use of Alexa Fluor^®^ 488 for studying the effect of structural parameters in the biodistribution of N-(2-Hydroxypropyl) methacrylamide (HPMA) copolymers, which is considered a versatile delivery platform for the early detection and intervention of orthopedic implant loosening (Wei et al., 2017). However, one of the main drawbacks of *in vivo* fluorescent live imaging is that labeling biotherapeutic agents with fluorescent probes can potentially compromise their biological activity (Cavaco et al., 2020; Hedegaard et al., 2018; Sánchez-Rico et al., 2017). In this regard, the European Medicines Agency (EMA) suggests that, before employing fluorescently labeled proteins for biodistribution studies, it is essential to ensure that the labeled compound retains its biological properties and activity comparable to those of the non-labeled material (European Medicine Agency, 2011). Although there exists a vast number of publications regarding PK or the biodistribution of purified peptides and proteins (Cronin and Yu, 2023; Diao and Meibohm, 2013; Sitte, 2016; van den Anker et al., 2018), similar reports on complex multipeptide drugs are scarce.

The hDLEs are mixtures of complex peptides with immunomodulatory properties. Transferon Oral^®^ (TO) is an hDLE composed of a mixture of low molecular weight peptides (<10 k Da) from 12 human protein clusters, as well as monomeric ubiquitin (Ub^1–76^ and Ub^1–74^) (Vallejo-Castillo et al., 2020). This batch-to-batch reproducible peptide mixture increases the survival of mice infected with HSV-1 by modulating the levels of IL-6, TNF-α, and IFN-ɣ (Medina-Rivero et al., 2014, 2016; Salinas-Jazmin et al., 2015). Additionally, TO modulates IFN-γ and CD4^+^ cells in herpes-zoster patients and improves the survival of septic pediatric patients by modulating C-reactive protein (CRP) and lymphocyte and neutrophil counts (Castrejón Vázquez et al., 2019; Estrada-Parra et al., 1998). It is worth mentioning that all the above results have been observed when TO is administered orally, indicating that its therapeutic targets may be located in the gastrointestinal tract or elsewhere, depending on the existence of an absorption phenomenon and the biodistribution pattern. To answer this question, the present work indirectly characterized the ADME profile (biodistribution) of the peptide components of TO using an imaging technique.

The description of the biodistribution profile of TO included three critical points: the fluorophore, the mice, and the verification that hDLE peptides maintain their biological activity after labeling. Firstly, the fluorophore Alexa Fluor 488 was chosen for this study because its pH stability range (pH 4–10) is compatible with the physiological pH of mice, including stomach pH, which is 4.0 under fasting conditions (McConnell et al., 2008; Panchuk-Voloshina et al., 1999). Besides, both hDLE peptides and Alexa Fluor 488 are hydrophilic molecules; thus, changes in their solubility after conjugation are not expected (Zanetti-Domingues et al., 2013). Secondly, we use nude mice to avoid fluorescence interference caused by hair (Hoshino et al., 2017). Thirdly, as previously mentioned, the physicochemical properties and biological functions of peptides or proteins can be altered, even obliterated, by the addition of a fluorophore (Cavaco et al., 2020). In this context, the murine model of HSV-1 infection was employed to assess the biological activity of hDLE-Alexa peptides, as this model is commonly used for releasing TO batches (Salinas-Jazmin et al., 2015). This model demonstrated that hDLE-Alexa peptides increase the mice’s survival (≥Δ40%) despite the addition of the fluorophore to their -NH_2_ groups, thus verifying that their behavior in the biodistribution assay is similar to that of the unlabeled peptides.

The biodistribution profile of hDLE peptides was investigated using female and male nude mice, administered both parenterally and enterally, to detect repetitive patterns. The IV route is the reference route for all pharmacokinetic studies because absorption phenomena are avoided in this route. For hDLE peptides, the IV route presented the fastest biodistribution profile compared to the rest of the routes, reaching maximum fluorescence in the cervical area of female and male mice at 15 min after administration. However, unlike other types of biopharmaceuticals, IV administration of peptides has the disadvantage that the elimination is favored through free filtration in the glomerulus, thus requiring a more frequent administration (Diao and Meibohm, 2013; Philippart et al., 2015). Furthermore, strain- and age-dependent dimorphism in Glomerular Filtration Rate (GFR) has been reported in mice (Tao et al., 2023) and our results suggest that GFR is higher in female Crl: Nu-Foxn1nu Nu/Nu nude mice than in males, since males exhibited a higher fluorescence biodistribution profile than females (1.26 × 10^9^ [p/s]/[μW/cm^2^] vs 5.12 × 10^8^ [p/s]/[μW/cm^2^]).

The other parenteral routes evaluated were SC, IP, and IM. A similar biodistribution profiles of hDLE were observed in SC and IP compared to IV administration: peptides migrated from the administration site to the neck and axillary region. However, two differences were observed in SC-administered female mice: an accumulation in the inguinal regions and long-lasting fluorescence (>180 min) in the ventral view. The intraperitoneal route showed differences in the biodistribution profile of hDLE peptides. On the other hand, after IP administration of hDLE in female mice, peptide migration from the inoculation site to other abdominal regions was observed, but little fluorescence was detected in the axillary area. In contrast, in male mice, SC and IP biodistribution were observed in the cervical and axillary areas. The hDLE peptides have a molecular weight less than 10 kDa, so they are absorbed through both blood capillaries and lymphatic vessels (Diao and Meibohm, 2013), and the biodistribution of hDLE peptides is influenced by the physiology of the administration site as well as by sexual dimorphism. One limitation of this study was that the synchronization of the menstrual cycle or ovariectomy in female mice was not considered. In this sense, it is possible that the role of hormones in regulating body fluids, the fat accumulation in the abdomen, and other physiological differences among mice sex, may be responsible for the observed variations in the SC and IP biodistribution profile of hDLE (Al Shoyaib et al., 2020; Erstad and Barletta, 2022; Small et al., 2022).

In the IM administration of hDLE, we observed a decrease in fluorescent signal at the administration site and the elimination of the peptides by glomerular filtration at 30 min in female mice and at 45 min in male mice. However, a biodistribution pattern towards the cervical and axillary areas was not observed. This difference can be explained because skeletal muscle contains a large amount of endoproteases and is responsible for the degradation of 26% of biotherapeutics, making it the second site with the highest rate of degradation after parenteral administration (Keizer et al., 2010). Furthermore, male mice have a greater muscle mass than female mice, favouring the degradation of hDLE (Erstad and Barletta, 2022). The IV, SC, and IP administration denote another limitation of using imaging techniques: the penetration capacity to detect the fluorescent signal, which depends on the tissue and the fluorophore used (Refaat et al., 2022). The study presented here detects fluorescence emission at an average depth of 2.6 mm, so the deeper the injection, the lower the detection rate. Therefore, *ex vivo* analysis is necessary to confirm the target organs (*vide infra*).

Oral administration is not a common route to deliver peptide-based drugs in living systems due to their poor bioavailability (Zhu et al., 2021). Notwithstanding, it is known that small peptides are more resistant to proteolytic cleavage and can cross the gastrointestinal barrier to reach the bloodstream (Räder et al., 2018; J. Wang et al., 2015b, 2015a). In this sense, the ORO pathway has been selected to successfully demonstrate the efficacy of TO as reported elsewhere (Hernández-Esquivel et al., 2018; Salinas-Jazmin et al., 2015). However, it is necessary to characterize the physiological mechanisms by which such absorption is carried out, as well as to identify accumulation sites to infer the mechanism of action of this complex mixture of peptides.

In 2020, the sequencing of TO peptides was published, and it was hypothesized that certain components, such as monomeric Ub, could exert their immunomodulatory function by interacting with the CXCR-4 receptors of the vagus nerve endings through the intestinal lumen (Vallejo-Castillo et al., 2020). The afferent fibers of the vagus nerve extend along all layers of the wall of the digestive tract but do not pass through the epithelial layer. Consequently, these fibers can only detect signals indirectly, either through the diffusion of compounds and metabolites from the microbiota or through other cells in the epithelium that transmit signals from the lumen (Bonaz et al., 2018). Similar to metabolites, peptides produced by gastrointestinal digestion regulate gut hormones through signaling pathways involving the GPCR family of receptors, making them novel targets for the regulation of various processes (Caron et al., 2017). Similarly, hDLE peptides are absorbed, like dietary nutrients, and enter the circulation, allowing their indirect detection by fluorescence in other organs.

Because hDLE peptides were observed to be orally absorbed, and this is the route used in efficacy studies, a secondary objective was to identify the organs and tissues immunologically relevant to the MOA of hDLE peptides, assuming the presence of a TMDD phenomenon. In this assay, hDLE-Alexa or an equivalent amount of the fluorophore alone was administered orally. Both hDLE peptides and Alexa are highly hydrophilic (Janiesch et al., 2015; Medina-Rivero et al., 2016), so they would be perfused into organs with high water content, such as the heart, lungs, liver, intestines, lymphatic system, and spleen. The imaging strategy employed would detect similar fluorescence in organs of animals administered hDLE-Alexa or Alexa; however, it would detect differences in organs and tissues where hDLE-Alexa peptides accumulated due to a TMDD effect, i.e., cell receptors that extensively interact with hDLE components. This hypothesis is supported by the results presented in this work, where hDLE peptides pass from the gastrointestinal tract to the bloodstream and lymphatic system, observing a statistically significant accumulation in axillary and cervical nodes. The accumulation of the hDLE peptides in the axillary nodes and heart in female mice and cervical and axillary nodes in male mice was confirmed by an *ex vivo* analysis. The accumulation of hDLE peptides in lymph nodes suggests that its biodistribution may be directed by a receptor, such as CXC-chemokine receptor 4 (CXCR4), which is described as the extracellular receptor to Ub and is associated with the homing of lymphoid cells to peripheral lymph nodes (Mendoza-Salazar et al., 2024; Su et al., 2006). The biodistribution of hDLE peptides to lymph nodes after ORO administration provides two advantages. First, the hDLE peptides directly interact with T lymphocytes, B lymphocytes, and dendritic cells, so their mechanism of action may involve direct modulation of these cells. Second, the biodistribution through the lymphatic system decreases the first-pass degradation effect (Yáñez et al., 2011), thereby prolonging the residence time of the peptides.

The other organ in which accumulation of hDLE peptides was observed, statistically significant in male mice and with a tendency for accumulation in females, was the heart. The heart is a bunch of vagus nerve endings, which are important regulators of the immune system (Abboud and Singh, 2017). While activation of the afferent system is recognized to promote inflammatory responses, activation of the efferent system promotes the secretion of acetylcholine (Ach), leading to a reduction in the secretion of proinflammatory cytokines, including IL-6, IL-1β, and TNF-α, by immune cells (Abboud and Singh, 2017; Bonaz et al., 2019; Koopman et al., 2016). As in the gastrointestinal tract, the anti-inflammatory effects of hDLE could be exerted through the vagus nerve endings in the heart through partial blockade of CXCR-4 by Ub (Hermann et al., 2008; Vallejo-Castillo et al., 2020). Furthermore, the sequence of the hDLE peptides suggests the possible existence of additional mechanisms of action. For example, hDLE contains hemoglobin peptides, and it was recently described that hemoglobin-derived peptides block the angiotensin-converting enzyme (ACE) (Outman et al., 2023). In this sense, blocking ACE decreases the production of angiotensin II, favoring greater activity of the vagus nerve and enhancing the release of acetylcholine and its consequent anti-inflammatory effects (Shanks and Ramchandra, 2021). The organs that showed the greatest fluorescence in the *ex vivo* analysis were the intestine, stomach, and liver, in both male and female mice. However, the difference between the fluorescence intensity of mice administered hDLE-Alexa and Alexa Fluor 488 was not statistically significant. Given that these are the organs most exposed to orally administered treatments, direct studies are required to confirm the accumulation of hDLE peptides in these organs.

It is important to note that the hDLE peptide accumulation findings presented in this work are based on the use of Alexa Fluor 488 as a control. Although the hDLE peptide mixture and the fluorophore are highly soluble, the difference in their sizes (≤10 kDa and 0.65 kDa, respectively) could affect their perfusion, introducing a bias into the hDLE TMDD patterns. Therefore, the hDLE accumulation results will be reinforced in subsequent studies using a biosynthetic hDLE-peptide mixture derived from the proteins reported by Vallejo-Castillo et al. (2020), as well as a negative control of scrambled peptides, both labeled with Alexa Fluor 488. Another option would be to use a dialyzed peptide extract derived from the buffy coats of a mammalian species other than humans. These controls, being peptide mixtures of similar size and physicochemical properties, will help identify TMDD patterns primarily governed by interactions between hDLE peptides and their potential receptors.

Our study demonstrates how oral administration of hDLE has advantages over intravenous administration. Although oral administration appears to result in lower absorption in male mice, hDLE levels in female mice administered orally are similar to those observed in male and female nude mice administered intravenously. However, oral administration provides sustained hDLE levels, whereas the intravenous route results in a rapid decline in fluorescence signal due to the high renal filtration rate of peptides (Diao and Meibohm, 2013). Given that hDLE is a mixture of small peptides, it is likely that different processes with varying rates contribute to maintain a constant level of signal: passive absorption into the bloodstream and lymphatic system, as well as through the use of specific receptors for the mixture’s components, such as CXCR4 and TLR2, or even receptors responsible for the nonspecific, unidirectional, low-energy transport of dietary peptides, such as oligopeptide transporter-1 (PepT-1) (Jia et al., 2025; Miao et al., 2023). Collectively, these mechanisms could delay the passage of DLE peptides into the general bloodstream and their subsequent renal filtration. Further studies are needed on the oral absorption pathways of DLE peptides and the apparent influence of sex.

## 5 Conclusion

The development of a complex multipeptide drug requires the application of high-throughput analytical methods to indirectly characterize its biodistribution profile. These profiles allow for a comprehensive characterization of the ADME process and the PK profile, as well as accumulation sites and potential target tissues. This work describes the biodistribution profile of Transferon Oral^®^ (hDLE), a complex peptide mixture, using *in vivo* fluorescence imaging in male and female nude mice. Parenteral and enteral routes of administration, despite the variability observed between sexes, indicate that hDLE peptides are absorbed from the site of administration, including the gastrointestinal tract, into cervical and axillary lymph nodes, as well as the heart. The hDLE peptides reach maximum biodistribution to target organs between 15 and 60 min and are eliminated around 180 min, depending on the administration route. Additionally, the biodistribution profile of hDLE identifies lymph nodes and the heart as the most relevant organs for studying the immunomodulatory effects of hDLE.

Understanding the MOA of the hDLE began with peptide sequencing; in this work, organs and tissues relevant to its biological effects have been identified. Further research can combine *in vivo* imaging with histological analysis in efficacy models, such as HSV-1 infection or allergic rhinitis, to determine which cells of the innate immune system have direct contact with hDLE peptides and whether the effect is also amplified by cellular pathways, in addition to the already known humoral pathways (IFN-ɣ, TNF-α, IL-6). Efficacy models will also help determine whether, during any pathology, hDLE peptides can exhibit novel TMDD patterns at the site of inflammation, potentially altering their ADME profile.

Finally, to the authors’ knowledge, this is the first study to use *in vivo* imaging to characterize the biodistribution profile of a multipeptide drug, so the strategy proposed here could be useful for advancing the pharmacological characterization and biocomparability of complex multipeptide drugs such as glatiramer acetate, bacterial lysates, and multipeptide vaccines, in addition to hDLEs.

## 6 Limitations

Among the strengths of this work is the proposed method for characterizing the biodistribution profile and TMDD phenomenon of a complex drug using fluorescence imaging. However, to better understand the results of this work, it is essential to state its limitations. Although the biodistribution profile of hDLE was evaluated *via* both parenteral and oral administration, only the latter was analyzed in depth (*ex vivo*). This is because the oral route is used in animal models, in which hDLE (Transferon Oral^®^) has demonstrated efficacy. In the *ex vivo* analysis, relevant organs were extracted according to the results of the corresponding biodistribution kinetics fluorescent pattern. In this regard, only the axillary and cervical lymph nodes were included in the *ex vivo* analysis. The identification of cervical and axillary lymph nodes was based on their macroscopic characteristics, anatomical position, and consistency (Supplementary Figure S1). This study did not include the identification of lymph node tissue based on histological analysis.

## Data Availability

The raw data supporting the conclusion of this article will be made available by the authors, without undue reservation.
