# Supplementary material for: Characterization of the biodistribution profile of a human Dialyzable Leukocyte Extract (hDLE) by in vivo fluorescence imaging: a strategy to infer the ADME profile of complex multipeptide drugs
Source: Front Pharmacol. 2026 Jan 12;16:1701647. doi: 10.3389/fphar.2025.1701647 (PMC12833350; doi:10.3389/fphar.2025.1701647)
Supplement: Supplementary file 1 [file DataSheet1.pdf]

Supplementary Material

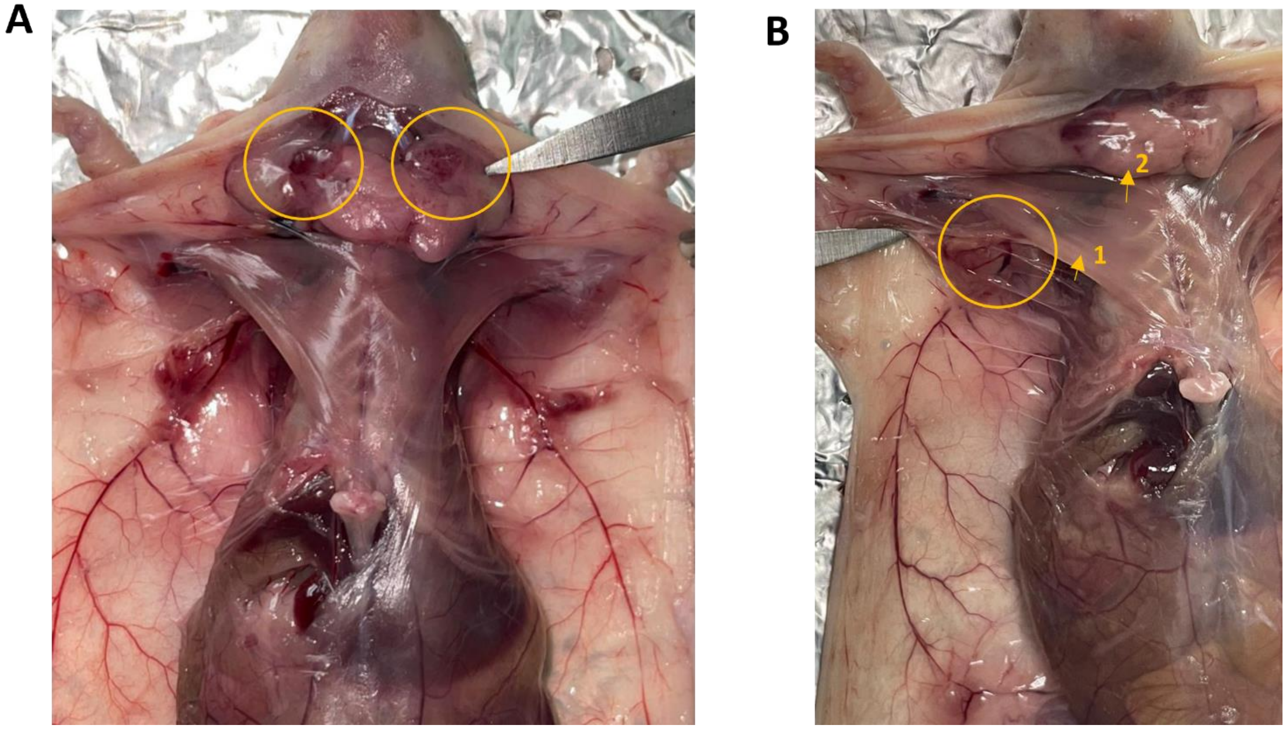

Figure S1. Anatomical identification of cervical and axillary lymph nodes in the nude mouse. The distribution and anatomical location of lymph nodes in the nude mouse are similar to those of the wild-type mouse. **A)** The group of cervical lymph nodes (yellow circles), formed by the mandibular lymph nodes (the largest), accessory mandibular and parotid, was located in the groove formed caudally by the pair of mandibular glands, easily identifiable, and rostrally by the rostral belly of the digastric muscle, the caudal end of the masseter muscle (mandibular attachment), and medially by the mylohyoid muscle. **B)** The main axillary and accessory axillary lymph nodes (yellow circle) were localized deep in subcutaneous fat, deep to the pectoral muscle (arrow 1). Dissection included cutting the triceps brachii and teres major muscles. Note the mandibular glands (arrow 2).

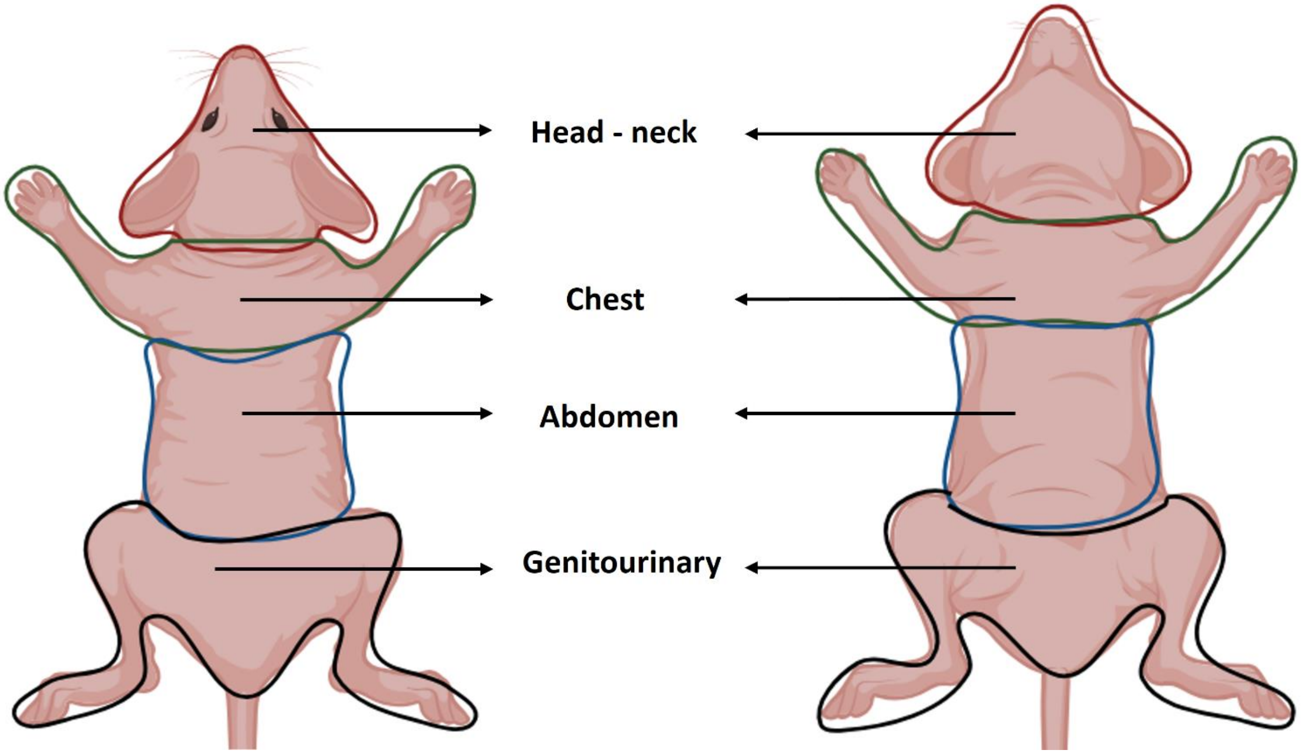

Figure S2. Regions of Interest (ROI), in which the images of the mice were divided for in vivo biodistribution analysis. The acquired images were split into four areas: head and neck (red), chest (green), abdomen (blue), and genitourinary (black) in both dorsal (left) and ventral views (right). Total radiant efficiency ([p/s/cm^2/sr]/[μW/cm^2]) was quantified in selected ROIs from dorsal and ventral views using Live Image® 4.3.1 software.

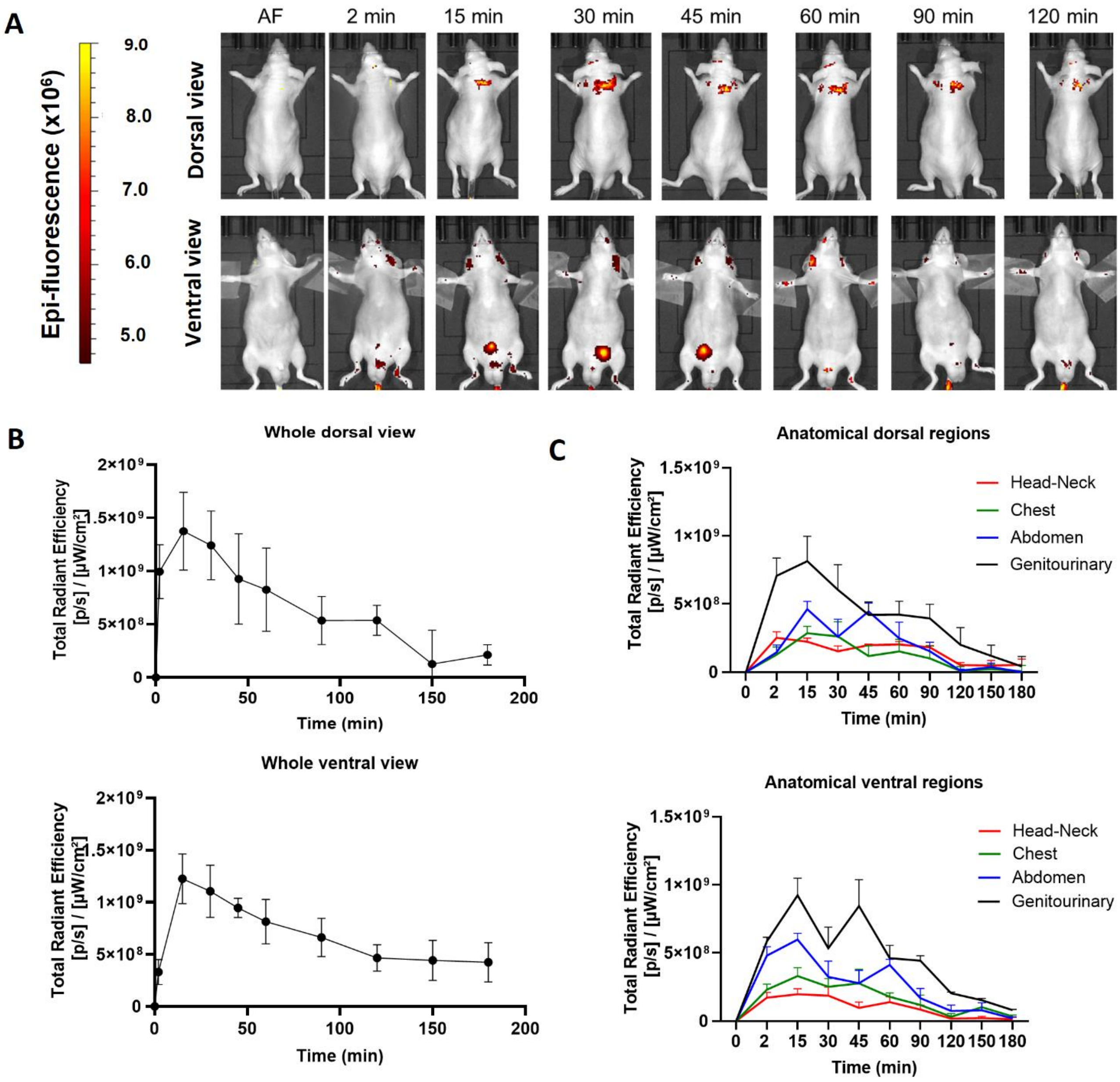

Figure S3. hDLE-Alexa peptides are distributed to the neck and genitourinary area of male Nu/Nu nude mice after IV administration. Four male animals were intravenously administered with hDLE-Alexa peptides, and epifluorescence was detected using an IVIS LUMINA XR system. **A)** Representative dorsal and ventral images of the same animal, where it is observed that peptides accumulate in the head-neck and genitourinary areas. **B)** Total body accumulation kinetics of the hDLE-Alexa peptides after intravenous administration in the four analyzed male mice; the peak of fluorescence is observed at 15 min in both dorsal and ventral views. **C)** Biodistribution kinetics of the hDLE-Alexa peptides in the four administered mice in each specific anatomical area (genitourinary, abdominal, chest, and head-neck); the genitourinary area exhibits the highest fluorescence levels in both dorsal and ventral views. Plots show the average total radiant efficiency of specific ROIs (whole or specific regions) over time (minutes) from dorsal and ventral images. Total Radial Efficiency ([p/s] / [µW/cm²]) was quantified in selected ROI from dorsal and ventral position images using the Live Image® 4.3.1 software. The standard error is presented in graphs B and C.

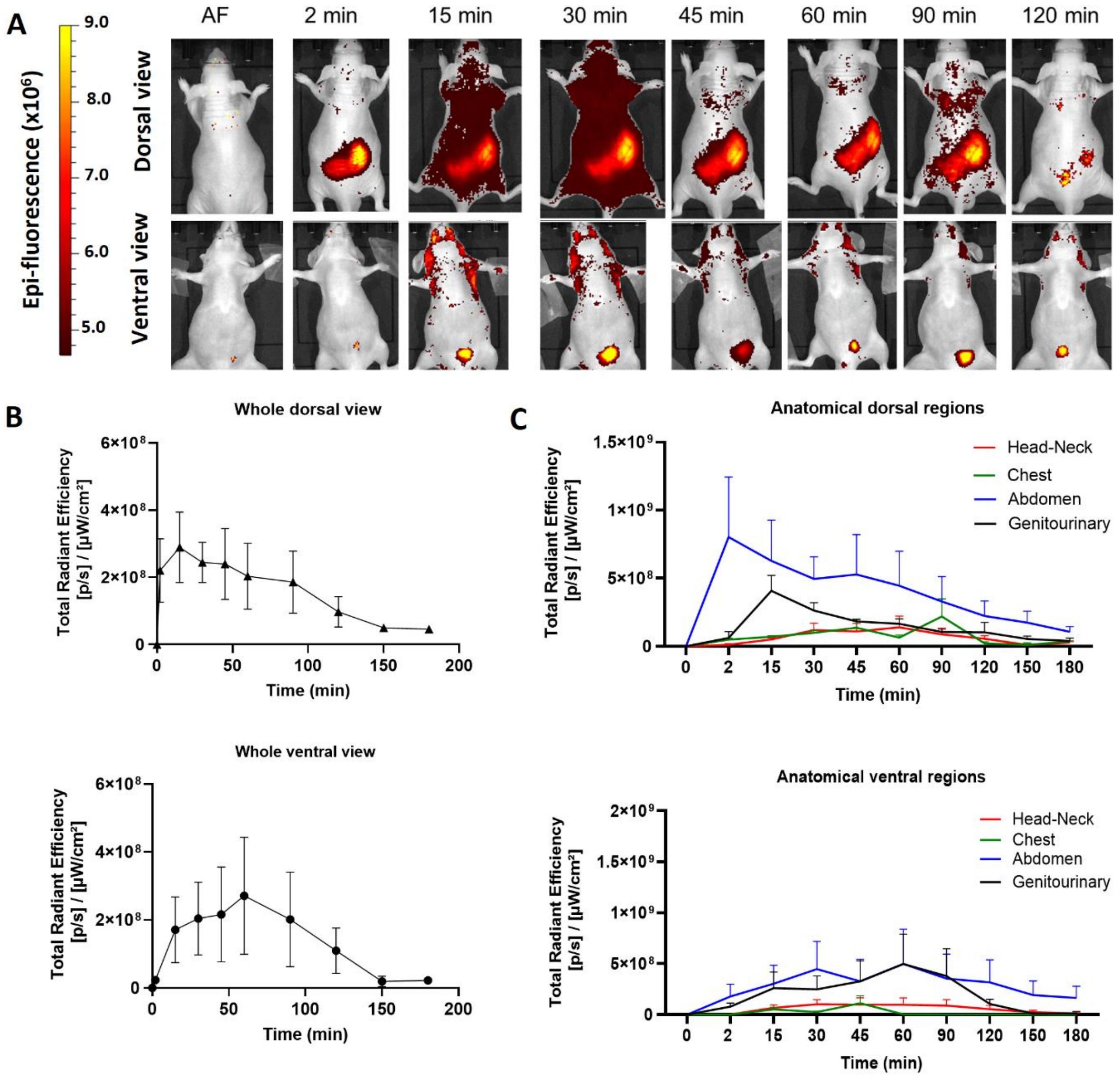

Figure S4. hDLE-Alexa peptides are widely biodistributed and accumulate in the head-neck and genitourinary area of Nu/Nu nude male mice after SC administration. Four animals were subcutaneously administered with hDLE-Alexa peptides in the back, and epifluorescence was detected using an IVIS LUMINA XR system. **A)** Representative dorsal and ventral images of the same animal in which a wide biodistribution of hDLE-Alexa peptides is observed in the dorsal view, whereas an accumulation in the head-neck and chest areas is observed in the ventral view. The two-minute dorsal view evinced the site of administration of the hDLE-Alexa peptides. **B)** Total body accumulation kinetics of the hDLE-Alexa peptides after SC administration in the 4 analyzed male mice; the maximum fluorescence is observed at 15 min in dorsal view and at 60 min in ventral view. **C)** Biodistribution kinetics of the hDLE-Alexa peptides in the 4 administered mice in each specific anatomical area (genitourinary, abdominal, chest, and head-neck). The abdomen area exhibited higher fluorescence in the dorsal view, and the genitourinary and abdomen in the ventral view. Plots show the average total radiant efficiency of specific ROIs (whole or specific regions) over time (minutes) from dorsal and ventral images. Total Radial Efficiency ([p/s] / [µW/cm²]) was quantified in selected ROI from dorsal and ventral position images using the Live Image® 4.3.1 software. The standard error is presented in graphs B and C.

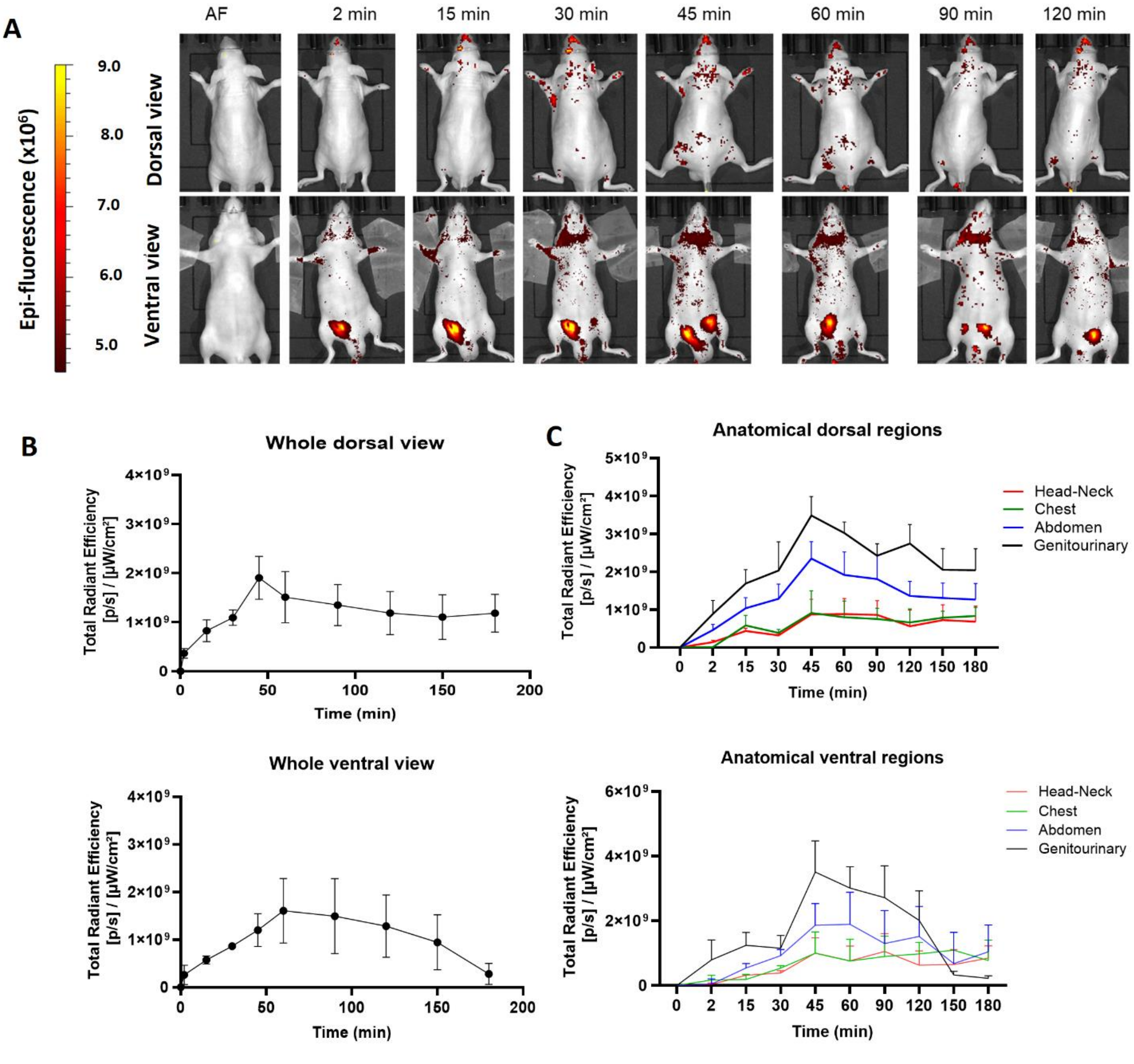

Figure S5. hDLE-Alexa peptides are extensively biodistributed in Nu/Nu nude male mice from the site of administration to the head-neck region after IP administration. The hDLE-Alexa peptides were intraperitoneally administered in four male Nu/Nu mice, and epifluorescence was detected using an IVIS LUMINA XR system. **A)** Representative dorsal and ventral images showing the biodistribution of the hDLE-Alexa peptides during 120 min; it is observed that the peptides are biodistributed from the administration site to the head-neck area. The two-minute ventral view evinced the site of administration of the hDLE-Alexa peptides. **B)** Total Radiant Efficiency plots of the dorsal and ventral accumulation fluorescence kinetics of the 4 administered mice; the maximum signal is observed around 45 min and 60 min in the dorsal and ventral view, respectively. **C)** Total Radiant Efficiency of the hDLE-Alexa peptides per specific anatomical area of the 4 administered mice (genitourinary, abdominal, chest, and head-neck) after IP administration. The region of greatest fluorescence is the genitourinary and abdominal areas in both ventral and dorsal views. The fluorescence in the administration site decreases from 2 min to 120 min, and a cyclic accumulation in the bladder is observed at 45 min, 90 min, and 120 min. Total Radial Efficiency ([p/s] / [µW/cm²]) was quantified in selected ROI from dorsal and ventral position images using the Live Image® 4.3.1 software. The plots represent the average radiant efficiency of all selected ROIs (whole or a specific region) over time, as determined from dorsal and ventral images of the four analyzed animals. The standard error is presented in graphs B and C.

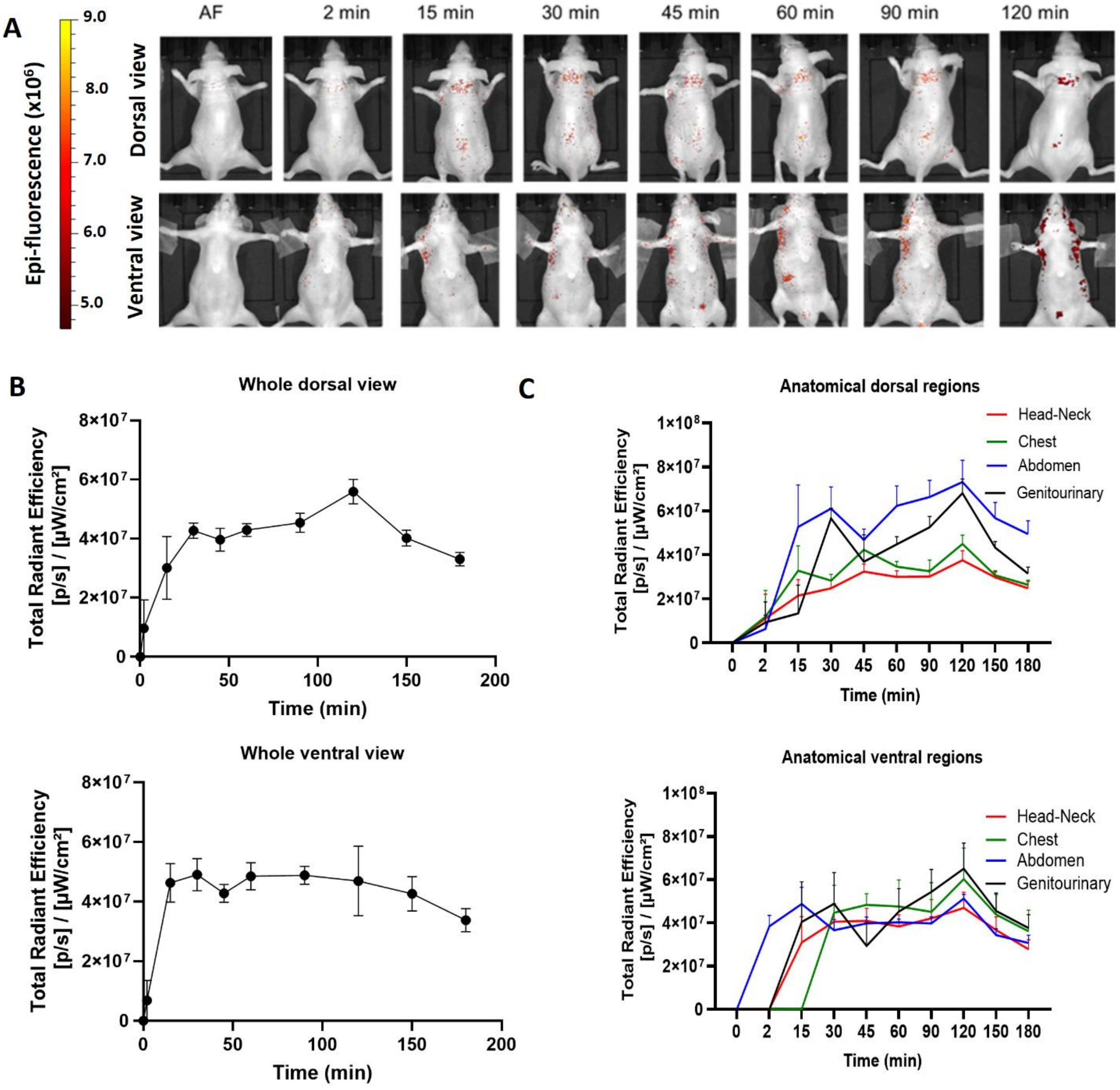

Figure S6. The hDLE-Alexa peptides are absorbed from the gastrointestinal tract and biodistributed to the head-neck and axillary area after ORO administration in male Nu/Nu nude mice. Four animals were administered with hDLE-Alexa peptides via ORO, and epifluorescence was detected using an IVIS LUMINA XR system. **A)** Representative dorsal (top) and ventral (bottom) images showing the biodistribution of the hDLE-Alexa peptides over a 120 min period. **B)** Global body accumulation kinetics of the hDLE-Alexa conjugate after ORO administration in male mice. Plots are the average of the total radiant efficiency of all selected Regions of Interest (ROI) vs time (min) from dorsal (top plot) and ventral (bottom plot) images. **C)** Biodistribution kinetics of the hDLE-Alexa conjugate in each specific anatomical area after ORO administration in male mice. Plots show the average total radiant efficiency of specific ROIs (genitourinary, abdominal, chest, and head-neck) over time (minutes) from dorsal (top plot) and ventral (bottom plot) images. Total Radial Efficiency ([p/s] / [µW/cm²]) was quantified in selected ROI from dorsal and ventral position images using the Live Image® 4.3.1 software. The standard error is presented in the B and C plots.

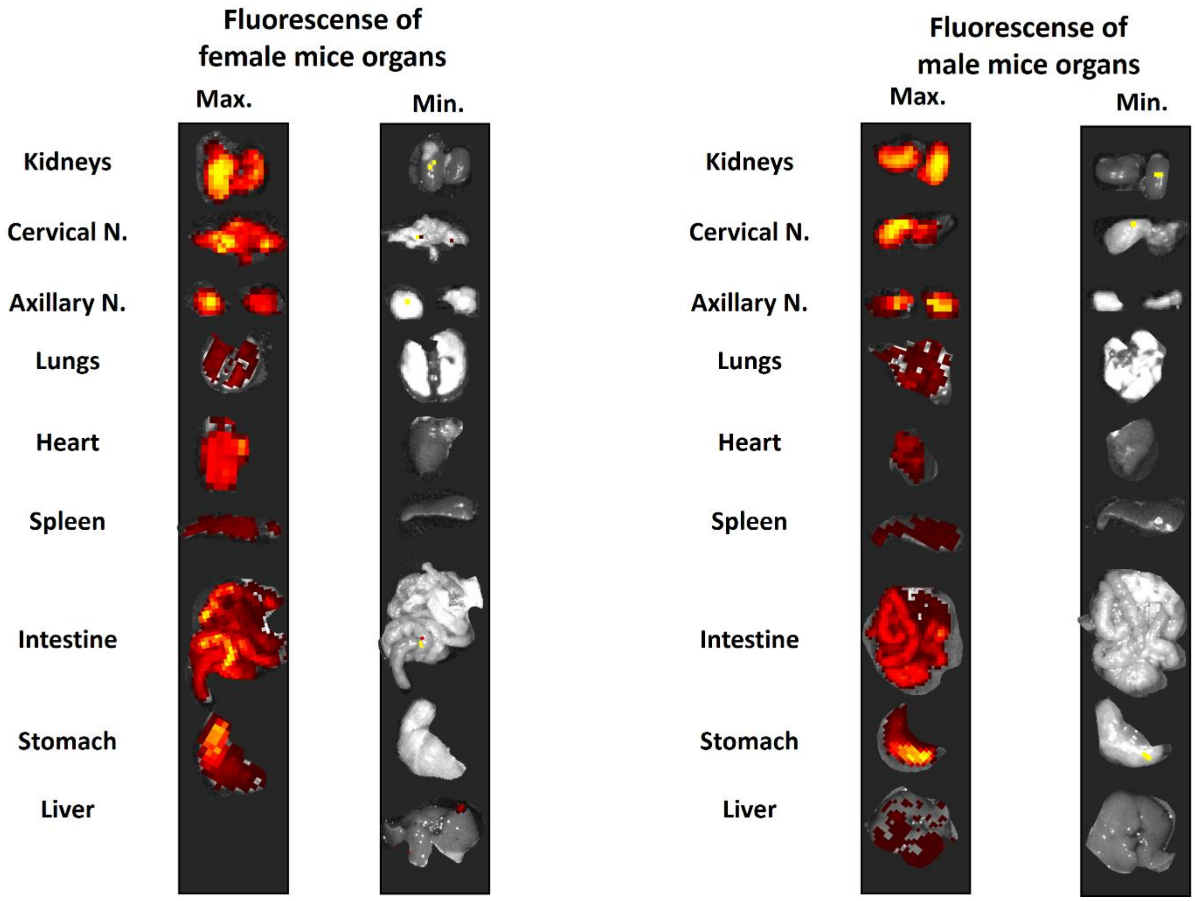

Figure S7. Identification of the organs of female and male mice administered with hDLE-Alexa after ORO administration. Nude mice were administered hDLE-Alexa via ORO and euthanized after 60 min. Then, the kidneys, cervical and axillary nodes, lungs, heart, spleen, intestine, stomach, and liver were extracted based on their macroscopic characteristics. Then, the fluorescence of each organ/tissue was acquired individually using the same parameters as in whole-animal kinetics (Max. fluorescence). To make the characteristics of the organs/tissues evident, the fluorescence signal was manually subtracted from the photographs (Min. fluorescence), and the organs were resized to a similar scale.
